# Tuning the tumor, trafficking the T cell: sIRE opens the door for solid tumor CAR T cells

**DOI:** 10.1016/j.omton.2026.201275

**Published:** 2026-06-30

**Authors:** Srijita Banerjee, Prachi Jalan, Bilguun Erkhem-Ochir, Govindarajan Srimathveeravalli, Prasad S. Adusumilli

**Affiliations:** 1 Thoracic Service, Department of Surgery, Memorial Sloan Kettering Cancer Center, New York, NY, USA; 2 Department of Mechanical and Industrial Engineering, University of Massachusetts, Amherst, MA, USA; 3 Institute for Applied Life Sciences, University of Massachusetts, Amherst, MA, USA; 4 Center for Cell Engineering, Memorial Sloan Kettering Cancer Center, New York, NY, USA

Effective chimeric antigen receptor (CAR) T cell therapy in solid tumors depends on successful tumor engagement, involving trafficking to tumor sites, intratumoral distribution, and sustained cytotoxic function within the tumor microenvironment. In solid tumors, T cells are frequently excluded from the tumor core and at times remain at the periphery or in the stroma, wherein the extracellular matrix restricts T cell migration within the tumor and limits direct interaction of the tumor and immune cells. This spatial separation represents a barrier to the efficacy of CAR T cells, as antigen recognition happens after efficient tumor access.^1^

To address this barrier, our team provided an important proof of concept that tumor-targeted, low-dose, nonablative radiation can function as a tumor-conditioning strategy to enhance CAR T cell therapy.^2^ We investigated CAR T cells that are already in use in the clinic^3^ in an orthotopic preclinical model of pleural mesothelioma: tumor-targeted radiation promoted antitumor efficacy by inducing a chemokine gradient (including CXCL9, CXCL10, and CXCL11) and by increasing levels of the chemokine receptor CXCR3 on CAR T cells. Augmented early tumor access increased infiltration, proliferation, and functional persistence of CAR T cells within the tumor. However, radiation-based tumor-microenvironment modulation is constrained by the limited number of doses that can be given to a tumor site; repeated doses of radiation kill CAR T cells.

Other locoregional approaches have been investigated as tumor-conditioning strategies to enhance adoptive cell therapy. Thermal modalities, such as microwave ablation, can remodel the tumor microenvironment and trigger immunogenic cell death, but they remain fundamentally indiscriminate, generating heat that destroys therapeutic CAR T cells alongside the tumor and precluding treatment after cell infusion.^4^ Although focused ultrasound techniques, such as with the sonogenetic EchoBack-CAR T platform, offer elegant spatial precision for T cell activation, they require complex genetic engineering and do not facilitate physical clearance of the tumor mass or chemokine recruitment provided by ablation.^5^

To improve tumor access to T cells, we chose irreversible electroporation (IRE), an established nonthermal ablation technique used clinically for solid tumors in the pancreas, liver, and prostate. By delivering high-voltage ultrashort electric pulses, IRE permanently permeabilizes the cell membrane to trigger cell death, without sustained temperature elevation, preserving the vasculature and adjacent critical structures. Beyond direct cytotoxicity, IRE-injured cells release damage-associated molecular patterns (DAMPs) and inflammatory chemokines that recruit immune cells into treated tissue (Figure 1). However, conventional IRE is nonselective and does not discriminate between cancer cells and tumor-infiltrating lymphocytes, limiting its repeated use. We developed selective IRE (sIRE) to address this limitation by exploiting fundamental biophysical differences in cell size and shape, as described in our recently published paper in *Clinical Cancer Research*.^6^ The larger, irregularly shaped cancer cells accumulate substantially higher transmembrane voltage than smaller, spherical T cells, under identical field conditions. By reducing the pulse width to limit transmembrane voltage escalation in T cells and increasing the number of pulses to maintain cancer cell lethality, we derived an optimized parameter set capable of differential cytotoxicity.

**Figure 1:**
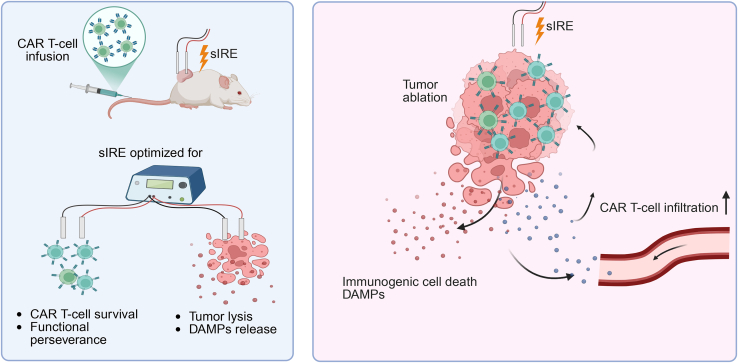
sIRE as a CAR T-cell-compatible tumor-ablative platform for solid tumor immunotherapy Following systemic CAR T cell infusion, locoregional sIRE is applied using optimized parameters that induce selective tumor cell lysis while sparing infiltrating CAR T cells. In contrast to conventional cytotoxic modalities, sIRE preserves CAR T cell viability and functional persistence (left). sIRE induces immunogenic cell death, leading to the release of DAMPs and tumor-derived chemokines, which enhance (i) CAR T cell trafficking and infiltration into the tumor and (ii) the development of memory responses against tumor cells (right). This immune-compatible tumor-ablation strategy positions sIRE as a repeatable locoregional tumor-conditioning platform that can be effectively integrated with cellular immunotherapies for solid tumors. Figure created in BioRender (https://app.biorender.com/illustrations/canvas-beta/69ef0e6d508b31e58a2faba6).

We further validated sIRE in a model of mesothelioma, which is an aggressive solid tumor. Finite element modeling confirmed that mesothelioma cells accumulate a 2.77-fold higher transmembrane voltage than CAR T cells, under similar field conditions. Our optimized sIRE pulse parameters (1,000 V/cm, 10-μs pulses, 200× pulses) lysed 64% of mesothelioma cells while preserving 86% of CAR T cells. In 3D hydrogel mimics, the ablation volume was 5.5-fold larger in mesothelioma hydrogels than in CAR T cell gels. We demonstrated that cancer cells injured by sIRE trigger release of DAMPs and chemokines, including a 10-fold increase in Hsp70 within 1 h and early increase of IL-1α and IL-1β, creating a chemotactic gradient that doubled CAR T cell transmigration.

When we translated this *in vivo*, a single sIRE pretreatment resulted in 4.7-fold more intratumoral CAR T cells, compared with infusion alone. Immunohistochemical analysis confirmed widespread intratumoral T cell distribution with sIRE, compared with peripheral-only distribution with CAR T cells alone or with conventional IRE. The combination of sIRE and CAR T cells accelerated tumor clearance, achieving complete eradication by day 15, versus day 26 with CAR T cells alone. Mice that had clearance of primary tumors subsequently had rejection of escalating intraperitoneal tumor rechallenge, confirming durable systemic immunity. Importantly, sIRE can be repeated up to three times without impairing CAR T cell cytotoxic function or phenotype, in contrast to conventional IRE. These results collectively distinguish sIRE as a translational approach, compared with existing locoregional tumor-conditioning approaches.

IRE has served purposes beyond tumor ablation and is recognized as a potent immune-priming tool through immunogenic cell death. In a model of hepatocellular carcinoma, IRE combined with GPC3-targeted CAR natural killer (NK) cells produced synergistic antitumor activity. IRE promoted immunogenic cell death and release of DAMPs, induced chemokines, increased NK cell infiltration, and increased the sensitivity of tumor cells to NK cell-mediated killing through increased intracellular reactive oxygen species.^7^ In a model of pancreatic cancer, IRE combined with allogenic CAR T cell therapy improved tumor control, compared with either modality alone.^8^ In patients with locally advanced pancreatic cancer, IRE combined with Vγ9Vδ2 T cell therapy (NCT03180437) showed prolonged survival, compared with IRE alone.^9^ In addition, the results of the IRE-IMMUNO study suggest that local IRE can also induce systemic antitumor immune activation in patients with prostate cancer.

By exploiting biophysical differences in cell size and shape to tune transmembrane voltage, our sIRE approach achieves differential cytotoxicity within a heterogeneous microenvironment, which is currently not possible with thermal, radiation-based, or conventional electroporation methods. sIRE can be administered repeatedly after CAR T cell infusion without compromising its function or phenotype. Additionally, it supports the development of durable immune memory capable of rejecting tumor rechallenge. These properties collectively define sIRE as a CAR T-cell-compatible tumor-conditioning platform in solid tumors. Immune activation alone is often transient and insufficient for durable tumor control, as the tumor microenvironment rapidly reestablishes immunosuppressive signaling; complementary strategies of combination therapy may be required.^10^

In summary, through a coordinated combination of biomechanical and bioengineering approaches, sIRE has demonstrated therapeutic benefits achieved by cancer cell lysis while preserving and augmenting CAR T cell efficacy.

## Acknowledgments

This research was funded in part through the NIH/NCI Cancer Center Support Grant P30 CA008748 (to Memorial Sloan Kettering Cancer Center). P.S.A.’s laboratory work is supported by grants from the National Institutes of Health (UG3CA290241, R01CA292664, R01CA235667, R01CA236615, and T32CA009501), the U.S. Department of Defense (CA200437), the Adolfo F. Sardiña Charitable Foundation, the Batishwa Fellowship, the Baker Street Foundation, the Joanne and John DallePezze Foundation, the Derfner Foundation, the Esophageal Cancer Education Fund, the Memorial Sloan Kettering Technology Development Fund, Mr. William H. Goodwin and Mrs. Alice Goodwin and the Commonwealth Foundation for Cancer Research, and the Center for Experimental Therapeutics of Memorial Sloan Kettering Cancer Center. P.S.A.’s laboratory receives research support from Novocure. G.S. acknowledges grant and funding support from the National Cancer Institute and the National Institute of Diabetes and Digestive and Kidney Diseases of the National Institutes of Health (R01CA236615 and R01DK129990), the U.S. Department of Defense (CDMRP PRCRP Award CA170630 and CA190888), and the Institute for Applied Life Sciences at the University of Massachusetts at Amherst.

## Declaration of interests

The authors report no relevant disclosures related to the work presented here. P.S.A. declares research funding from Novocure; is a Scientific Advisory Board Member and/or Consultant for Affyimmune Therapeutics, Bio4t2, Carisma Therapeutics, Century Therapeutics, Orion Pharma, Outpace Bio, Pluri-biotech, and Verismo Therapeutics; has patents, royalties, and intellectual property on mesothelin-targeted CAR and other T cell therapies, an issued patent method for detection of cancer cells using virus, and pending patent applications on PD1 dominant negative receptor, a wireless pulse-oximetry device, and on an *ex vivo* malignant pleural effusion culture system. Memorial Sloan Kettering Cancer Center has previously licensed intellectual property related to mesothelin-targeted CARs and T cell therapies to ATARA Biotherapeutics and had associated financial interests. G.S. holds equity in Aperture Medical and Kunam Medical and patents related to use of IRE for cancer diagnosis and treatment.
